# Mahuang Fuzi Xixin Decoction Ameliorates Allergic Rhinitis in Rats by Regulating the Gut Microbiota and Th17/Treg Balance

**DOI:** 10.1155/2020/6841078

**Published:** 2020-05-25

**Authors:** Xiao Liang, Chang-Shun Liu, Xiao-Han Wei, Ting Xia, Fei-Long Chen, Qing-Fa Tang, Meng-Yue Ren, Xiao-Mei Tan

**Affiliations:** ^1^School of Traditional Chinese Medicine, Southern Medical University, Guangzhou, China; ^2^Guangdong Provincial Key Laboratory of Chinese Medicine Pharmaceutics, Guangzhou, China; ^3^Guangdong Provincial Engineering Laboratory of Chinese Medicine Preparation Technology, Guangzhou, China

## Abstract

Mahuang Fuzi Xixin Decoction (MFXD), a Chinese traditional herbal formulation, has been used to treat allergic rhinitis (AR) in China for centuries. However, the mechanism underlying its effect on AR is unclear. This study investigated the mechanism underlying the therapeutic effects of MFXD on AR. Ovalbumin-induced AR rat models were established, which were then treated with MFXD for 14 days. Symptom scores of AR were calculated. The structure of the gut microbiota was analyzed by 16S rRNA gene sequencing and qPCR. Short-chain fatty acid (SCFA) content in rat stool and serum was determined by GC-MS. Inflammatory and immunological responses were assessed by histopathology, ELISA, flow cytometry, and western blotting. Our study demonstrated that MFXD reduced the symptom scores of AR and serum IgE and histamine levels. MFXD treatment restored the diversity of the gut microbiota: it increased the abundance of Firmicutes and Bacteroidetes and decreased the abundance of Proteobacteria and Cyanobacteria. MFXD treatment also increased SCFA content, including that of acetate, propionate, and butyrate. Additionally, MFXD administration downregulated the number of Th17 cells and the levels of the Th17-related cytokines IL-17 and ROR*γ*t. By contrast, there was an increase in the number of Treg cells and the levels of the Treg-related cytokines IL-10 and Foxp3. MFXD and butyrate increased the levels of ZO-1 in the colon. This study indicated MFXD exerts therapeutic effects against AR, possibly by regulating the gut microbial composition and Th17/Treg balance.

## 1. Introduction

Allergic rhinitis (AR), an immunoglobulin E- (IgE-) mediated respiratory allergic disease, is characterized by nasal itching, congestion, and sneezing [1]. The incidence of AR is constantly rising, almost reaching an epidemic level [2]. AR has multiple comorbidities, such as asthma and headache, thereby deteriorating the quality of life of patients [3]. In clinical setting, AR is mainly treated with inhaled corticosteroids, antihistamines, and leukotriene-receptor antagonists. However, these drugs can only provide temporary relief, and the symptoms will soon relapse after drug withdrawal [4]. The use of traditional herbal medicines for AR has been widely accepted in China and the surrounding areas owing to their good efficacy and few side effects.

Mahuang Fuzi Xixin Decoction (MFXD), which is described in Zhongjing Zhang's *Shanghan Lun*, is a traditional polyherbal mixture composed of Mahuang (the dried stems of *Ephedra sinica Stapf*, *Ephedra equisetina* Bunge, or *Ephedra intermedia* Schrenk & C.A. Mey.), Fuzi (the lateral root of *Aconitum carmichaelii* Debeaux), and Xixin (the root and rhizome of *Asarum heterotropoides f. mandshuricum* (Maxim) Kitag.). Mahuang, Fuzi, and Xixin have anti-inflammatory effects [5, 6]. MFXD is considered an effective intervention for AR and inflammation [7]. In previous studies, we identified 37 bioactive ingredients from MFXD and potential targets related to AR by the network pharmacology method [8]. Moreover, we identified the chemical profile and nine main chemical compounds of MFXD by UPLC-MS/MS [9]. Recent studies have suggested that respiratory allergic diseases are strongly related with disturbances of gut microbiota [10]. As MFXD is administered orally, its interaction with gut microbiota is inevitable.

Gut microbiota is considered beneficial because it provides protection from pathogens, nutrition, metabolic benefits, and immune system support [11]. However, dysbiosis of gut microbiota markedly affects microbiota-host interactions and inhibits the host immune system [12, 13]. Changes in lifestyle, disease, use of drugs, or diet can impact gut microbiota composition [14]. Evidence has shown that probiotic supplementation can modulate immune responses in AR by restoring gut microbiota dysbiosis [15, 16]. Gut microbiota ferment fiber and produce metabolites, such as short-chain fatty acids (SCFAs) (e.g., acetate, propionate, and butyrate), lipids, vitamins, and bile acids [17]. These metabolites extensively affect intestinal immune homeostasis, affect the immune system directly or indirectly, and protect the host from developing allergic diseases [18]. SCFAs have been considered potential mediators involved in the effects of gut microbiota on the intestinal immune function. SCFAs, particularly butyrate, can enhance Treg production and inhibit Th17 differentiation through the peroxisome proliferator-activated receptor gamma pathway [19]. Thus, Th17 and Treg cells are key cell subsets connecting gut microbiota and the immune system [20, 21]. In summary, gut microbiota and its metabolites might provide a novel understanding of MFXD.

In the present study, we investigated the effect of MFXD on gut microbial composition and Th17/Treg balance and further examined the therapeutic mechanisms of MFXD. This study might provide a new insight into the immunomodulatory effects of MFXD on AR.

## 2. Materials and Methods

### 2.1. Materials

Mahuang, Fuzi, and Xixin decocting pieces were obtained from Kangmei Pharmaceutical Co., Ltd. (Guangzhou, China). The voucher specimens (No. 160350561) were deposited in our laboratory. Ovalbumin (OVA) was purchased from Sigma (Missouri, USA). Aluminum hydroxide was purchased from Damao Chemical Reagent Factory (Tianjin, China). IgE and histamine (HIS) ELISA kits were purchased from Nanjing Jiancheng Bioengineering Institute (Nanjing, China). Rat IL-10, IL-17, IL-1*β*, and IL-23 ELISA kits were purchased from Cusabio Biotech Co., Ltd. (Wuhan, China). Rat peripheral blood lymphocyte separation medium kit was purchased from Solarbio (Beijing, China). Rat-FITC-anti-CD4, Rat-Cy3-anti-CD25, Rat-APC-anti-Foxp3, and Rat-Alexa Flour-488 IL-17 were purchased from eBioscience (California, USA). Anti-rat ZO-1 antibody was purchased from Abcam (Cambridge, UK). The other chemical reagents used were of analytical grade.

### 2.2. Preparation of MFXD

MFXD consisted of Mahuang, Fuzi, and Xixin at a ratio of 2 : 3 : 1. Mahuang was immersed in distilled water (15 times the total weight) for 30 min and boiled for 20 min. Next, Fuzi and Xixin were added to the suspension, which was then simmered for another 90 min. Filtrates were concentrated to 1.52 g/mL by rotary evaporation.

### 2.3. Animal Experiments

Specific pathogen-free male Wistar rats (160 ± 20 g) were obtained from the Experimental Animal Center of Southern Medical University (Guangzhou, China) and housed in a specific pathogen-free animal laboratory with a relative humidity of 60% and a temperature of 25°C under a 12 h light/dark cycle. All rats were provided specific pathogen-free food and water *ad libitum* and acclimated for one week. The study was approved by the Institutional Animal Care and Use Committee of Southern Medical University (No. L2018130).

The animal experiment was conducted by referencing Ren's design with minor modification [9]. The whole experiment lasted for 35 days. AR rat models were induced with OVA. Briefly, 0.3 mg OVA and 30 mg aluminum hydroxide were dissolved in 1 mL of saline solution, and this mixture was intraperitoneally injected to the rats every other day (sensitization stage). After that, the rats were provoked with administration of 50 *μ*L of 5% OVA solution by a micropipette into each nostril once daily for 7 days. Establishment of AR models was confirmed by observing the behaviors of rats and detecting serum IgE level.

Normal rats were used as the control group (*n* = 6). AR rats were randomly divided into four groups (*n* = 6): the AR model (sterile water), MFXD (7.6 g/kg), loratadine (1 mg/kg), and sodium butyrate groups (200 mg/kg). The dose of MFXD in the study is equivalent to 84 g, recorded by *Shanghan Lun*, according to the conversion by body surface area. The treatments were orally administered once daily from day 22 to day 35. The rats were intranasally dripped every other day to maintain nasal sensitization during treatment.

Nasal symptom scores were measured on day 35. The rats were placed in separate cages under observation of 30 min. The frequencies of nasal scratching, sneezing, and rhinorrhea were recorded and calculated (Supplementary table S1). Next, blood samples were collected from the rats via the abdominal aorta under anesthesia. Sera were obtained through centrifugation of blood samples (3500 rpm, 15 min at 4°C) and then stored at −20°C. Nasal and colonic mucosa samples were collected for histochemical analysis. The lung tissue was homogenized and centrifuged. Then, the supernatants were used for further measurement of lung cytokine levels. Colon tissue and stool samples were collected and immediately stored at −80°C.

### 2.4. Histochemical Analysis

Nasal mucosa was fixed with 4% paraformaldehyde, dehydrated, and embedded in paraffin. The tissues were cut into 4 *μ*m thick sections and stained with hematoxylin-eosin (HE) and Periodic Acid-Schiff (PAS). Histopathological changes were assessed and photographed by an optical photomicroscope.

Colonic tissue sections were dewaxed, rehydrated, and retrieved antigen. After washing with phosphate-buffered saline (PBS), the sections were incubated with anti-rat ZO-1 antibody at 4°C overnight, followed by incubation with secondary antibodies at 37°C for 60 min. After washing with PBS, staining was coupled three times, and 30-diaminobenzidine was added for color development. All slices were counterstained with hematoxylin at 25°C for 1 min. Microscopic images were obtained with a light microscope.

### 2.5. ELISA Tests

The levels of IgE, HIS, IL-10, and IL-17 in serum and IL-1*β* and IL-23 in lung tissues were detected using ELISA kits, according to the manufacturers' instructions.

### 2.6. 16S rRNA and qPCR Microbiome Analysis

Total genomic DNA in stool samples was extracted using E.Z.N.A. Stool DNA Kit. The V4 region of the eukaryotic ribosomal RNA gene was amplified by PCR using the primers 341F (CCTACGGGNGGCWGCAG) and 806R (GGACTACHVGGGTATCTAAT). Amplicons were extracted from 2% agarose gels, purified by AxyPrep DNA Gel Extraction Kit, and quantified by QuantiFluor-ST. The concentration and purity of DNA were measured with a spectrophotometer.

16S rRNA high-throughput sequencing was performed on an Illumina MiSeq2500 commercial platform. Purified amplicons were pooled in equimolar concentrations and paired-end sequenced (2 × 250) on an Illumina platform according to the standard protocols. Reads were assembled by FLASH (version 1.2.11) and filtered by QIIME (version 1.9.1). After quality filtering and chimera removal, clean data in each sample were clustered into operational taxonomic units (OTUs) with 97% similarity identity using UPARSE pipeline. The phylogenetic affiliation of each 16S rRNA gene sequence was assigned by RDP classifier. Bioinformatics analysis will be carried out with the obtained sequencing data.

Based on the sequencing data, the bacteria selected for qPCR are well-known bacteria in the intestines. *Enterococcus* spp., *Butyricicoccus pullicaecorum*, and *Lactobacillus group* belong to Firmicutes. The *Bacteroides fragilis* group belongs to Bacteroidetes. qPCR assays were carried out in a 96-well optical plate on a LightCycler® 480 Real-Time PCR System (Roche Diagnostics, Basel, Switzerland). All assays were performed twice. The reaction mixtures consisted of TaKaRa Premix Taq (10 *μ*L), template DNA (2 *μ*L), 10 *μ*M forward primer (0.4 *μ*L), 10 *μ*M reverse primer (0.4 *μ*L), and ddH_2_O (7.2 *μ*L). The copy number of target DNA was measured by serially diluting standards running on the same plate. Bacterial quantity was presented as log10 bacteria per gram of stool.

### 2.7. SCFA Level Determination

Stool samples were diluted in water, ultrasonically mixed, and centrifuged at 15000 rpm and 4°C for 10 min. The supernatant (0.5 mL), anhydrous sodium sulfate (0.3 g), 50% sulfate acid (10 *μ*L), and ether (1 mL) were added into a 4 mL EP tube and vortex-mixed. The mixture was then centrifuged at 4000 rpm and 4°C for 20 min. The serum samples (100 *μ*L) were added methanol (200 *μ*L), mixed, and centrifuged at 10000 rpm and 4°C for 10 min. The supernatant was analyzed by GC-MS to determine SCFA content.

### 2.8. Flow Cytometry Detection

Peripheral blood mononuclear cells were isolated from heparinized blood samples based on the instruction provided in rat peripheral blood lymphocyte separation kit. Isolated cells were washed three times with PBS and subjected to flow cytometry. PBMCs were obtained, and their surfaces were stained with CD4-FITC and CD25-Cy3. Subsequently, the cells were stained with an APC anti-rat Foxp3 or an Alexa Flour-488 IL-17 staining kit, according to the manufacturers' instructions. CD4+IL-17+ Th17 cells and CD25+Foxp3+ Treg cells were detected using a flow cytometer assay.

### 2.9. Western Blotting Analysis

For Foxp3 and ROR*γ*t analyses, total proteins from colon tissue were extracted using a radio-immunoprecipitation lysis solution and then measured with a bicinchoninic acid protein assay kit. Total proteins (50 *μ*g) were subjected to 10% SDS-PAGE and then transferred onto PVDF membranes. The membranes were then blocked in 5% nonfat milk solution, incubated with the appropriate concentration of primary antibodies against ROR*γ*t or Foxp3 overnight at 4°C, and then incubated with secondary antibody. Finally, protein bands were visualized using an enhanced chemiluminescence detection kit and quantified by optical density using the ImageJ software. The results are expressed as a ratio to *β*-actin.

### 2.10. Statistical Analysis

All data are presented as the mean ± SD. Statistical analyses were performed using one-way analysis of variance, followed by Tukey's *post hoc* test. Statistical significance was accepted at *P* values of less than 0.05.

## 3. Results

### 3.1. MFXD Treatment Alleviated the Symptoms of AR in Rats

**Figure 1:**
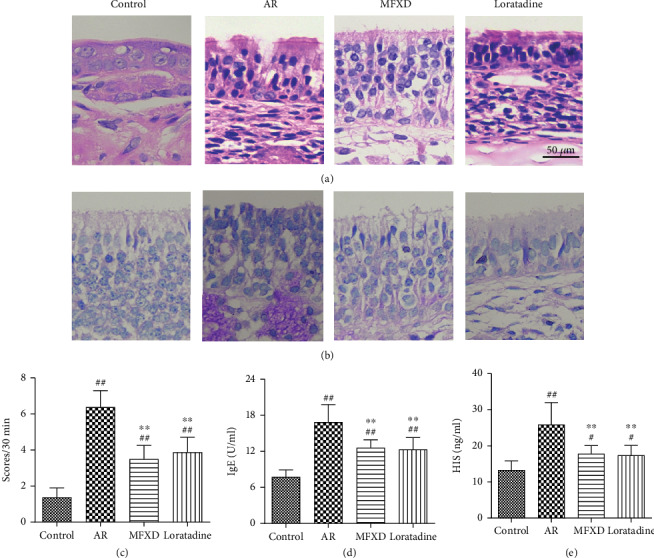
MFXD ameliorates OVA-induced AR in rats. (a) HE staining (400x magnification). (b) PAS staining (400x magnification). (c) Nasal symptom scores were counted for 30 min after the last nasal instillation. IgE (d) and HIS (e) levels in serum were determined by ELISA. Data are presented as the means ± SD. ^#^*P* < 0.05 and ^##^*P* < 0.01 vs. the control group; ^∗^*P* < 0.05 and ^∗∗^*P* < 0.01 vs. the AR group.

The effect of MFXD on AR was evaluated by histopathological changes in nasal mucosa, nasal symptom scores, and serum IgE and HIS levels. HE and PAS staining clearly revealed overall damage to the surface epithelium, disruption of the cryptal glands, and infiltration of inflammatory cells in the AR group, but these changes were reversed by MFXD treatment (Figures 1(a) and 1(b)). Nasal symptom scores in the AR group increased compared with those in the control group (*P* < 0.05), but treatment with MFXD markedly decreased these scores (Figure 1(c)). Moreover, the levels of IgE and HIS in the AR group were higher than those in the control group, whereas treatment with MFXD decreased the IgE and HIS levels (Figures 1(d) and 1(e)). Furthermore, the effect of MFXD on AR was similar to that of the clinical drug loratadine.

### 3.2. MFXD Restored the Structure of Gut Microbiota in AR Rat Models

**Figure 2:**
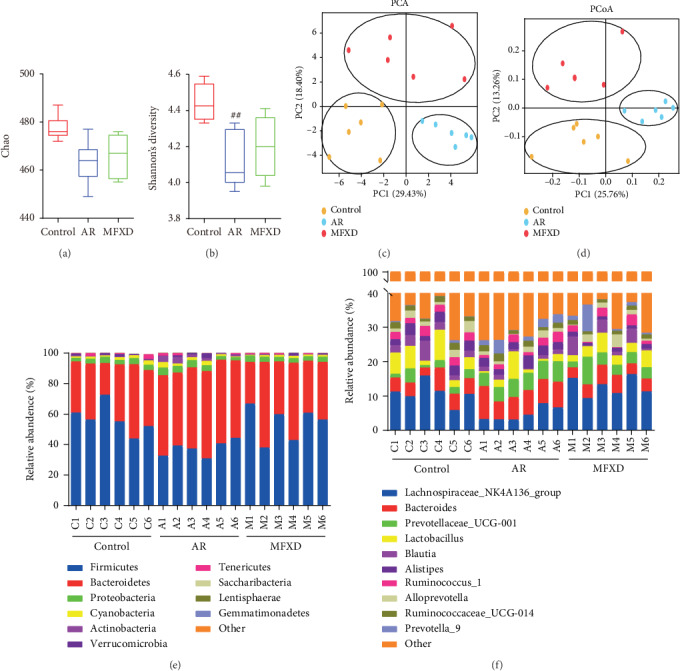
MFXD treatment modulated the structure of gut microbiota in AR rats. To assess the effects of MFXD on gut microbiota, 16S rRNA sequencing of rat fecal DNA was performed. Alpha-diversity analysis by Chao indexes (a) and Shannon indexes (b). (c) PCA of gut microbiota of rats from the control, AR, and MFXD groups. (d) PCoA of gut microbiota based on weighted UNIFRAC metrics indicated the different *β*-diversity of gut microbiota. (e) Relative abundance of different bacterial phyla in each group. (f) Relative abundance of different bacterial genera in each group. Data are presented as the means ± SD. ^#^*P* < 0.05 and ^##^*P* < 0.01 vs. the control group; ^∗^*P* < 0.05 and ^∗∗^*P* < 0.01 vs. the AR group.

We studied the diversity of the gut microbiota, and the results showed that Chao and Shannon indexes were decreased in the AR group compared to those of the control group. Administration of MFXD increased both indexes (Figures 2(a) and 2(b)). Principal component analysis (PCA) and principal coordinate analysis (PCoA) clearly distinguished the control, AR, and MFXD groups (Figures 2(c) and 2(d)). These results suggested that gut microbiota structure changed in response to MFXD treatment.

Taxon-based analysis showed marked differences in gut microbial composition at both the phylum and genus levels. At the phylum level, Firmicutes, Bacteroidetes, Proteobacteria, Actinobacteria, and Cyanobacteria showed the highest relative abundance in all samples (Figure 2(e)). However, in the AR group, the relative abundance of Firmicutes (37.96%) and Actinobacteria (0.38%) decreased, whereas that of Proteobacteria (3.61%) and Cyanobacteria (1.84%) increased (the abundance of Firmicutes, Actinobacteria, Bacteroidetes, Proteobacteria, and Cyanobacteria in the control group was 58.81%, 1.27%, 34.10%, 3.14%, and 1.66%, respectively). Administration of MFXD reversed these changes (the abundance of Firmicutes, Actinobacteria, Bacteroidetes, Proteobacteria, and Cyanobacteria was 50.67%, 0.40%, 43.59%, 3.17%, and 1.10%, respectively). At the genus level, there was a difference between the three groups (Figure 2(f)). The relative abundance of *Anaerotruncus*, *Butyricicoccus*, *Lachnospiraceae*, and *Ruminococcaceae* significantly increased after MFXD treatment. The abundance of *Bacteroides*, *Bifidobacterium*, *Enterococcus*, and *Erysipelotrichaceae* significantly decreased after MFXD treatment (Table 1).

The levels of fecal bacteria were further verified by qRT-PCR. Bacterial copy number values were transformed into logarithmic values before analysis. Quantities of gene copies of *Lactobacillus group* and *Butyricicoccus pullicaecorum* in the AR group were lower than those in the control group, *Bacteroides fragilis* group, and *Enterococcus* spp. and were markedly increased in the AR group, compared with the control group. However, after treatment with MFXD, this trend was reversed (Table 2). This result was consistent with the 16S rRNA sequencing results.

### 3.3. MFXD Promoted Fecal SCFA Levels and Gut Integrity

**Figure 3:**
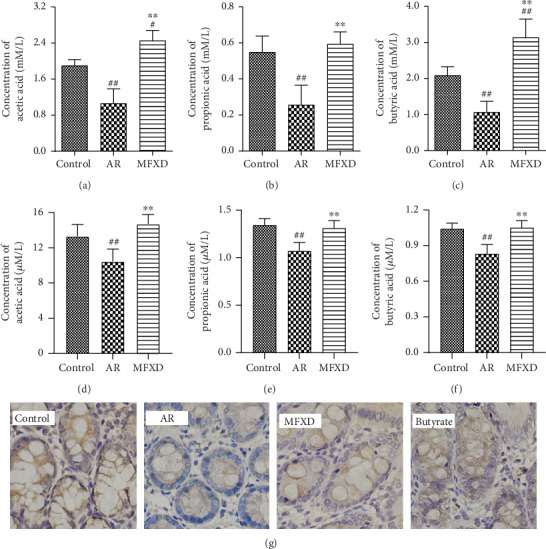
MFXD promoted fecal SCFA levels and gut integrity. Fecal concentrations of acetic acid (a), propionic acid (b), butyric acid (c), serum concentrations of acetic acid (d), propionic acid (e), and butyric acid (f) in rats were measured by GC-MS. (g) Expression of the tight junction proteins ZO-1 was detected by immunohistochemistry. Data are presented as the means ± SD. ^#^*P* < 0.05 and ^##^*P* < 0.01 vs. the control group; ^∗^*P* < 0.05 and ^∗∗^*P* < 0.01 vs. the AR group.

Considering the important role of SCFAs in microbiota-host interactions, we measured whether MFXD affects SCFA levels in AR rats. Results showed that the serum levels of SCFAs are lower than those in the feces. SCFA levels in the feces and serum were lower in the AR group than in the control group (*P* < 0.01). MFXD treatment significantly increased the levels of SCFAs in both the feces and serum. Acetic, propionic, and butyric acids showed the same trend and returned to normal levels after MFXD treatment (Figure 3).

In addition, the expressions of ZO-1 were downregulated in the AR group, which indicates the damage of gut integrity. However, MFXD reversed these changes (Figure 3(g)). Butyrate, one of important SCFAs in the gut, was found to repair the gut integrity after treatment. These results indicate that MFXD promoted the levels of fecal SCFAs which may improve the gut integrity of colon.

### 3.4. MFXD Regulated the Serum and Lung Levels of Cytokines in Rats

**Figure 4:**
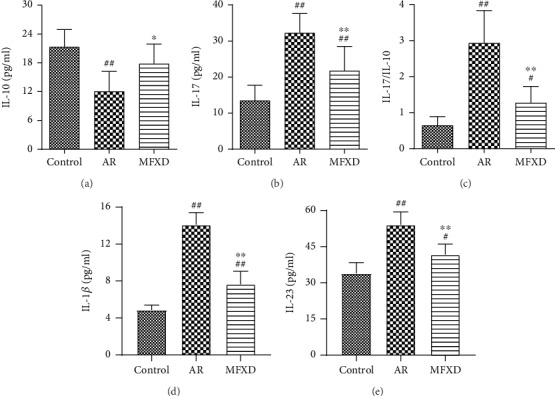
MFXD regulated the serum and lung levels of cytokines in rats. The serum levels of IL-10 (a), IL-17 (b), and IL-17/IL-10 ratio (c) in all groups. (d) IL-1*β* and (e) IL-23 levels in rat lung tissues. Data are presented as the means ± SD. ^#^*P* < 0.05 and ^##^*P* < 0.01 vs. the control group; ^∗^*P* < 0.05 and ^∗∗^*P* < 0.01 vs. the AR group.

IL-10 and IL-17 are the major cytokines of Treg and Th17 cells. ELISA tests showed that the levels of IL-10 and IL-17 in the AR group decreased and increased, respectively, compared with those in the control group (*P* < 0.01). However, after treatment with MFXD, changes in the levels of IL-10 and IL-17 were noticeably reversed (Figures 4(a) and 4(b)). Furthermore, the ratio of Th17/Treg cells in the AR group increased compared with that in the control group, whereas MFXD treatment markedly decreased this ratio (Figure 4(c)).

Moreover, the level of IL-1*β* and IL-23 in lung tissues was significantly elevated in the AR group in comparison with the control group. However, the levels of these cytokines were markedly declined after treatment with MFXD. These results indicate that MFXD is capable of alleviating inflammatory response in the lung tissues of AR rats (Figures 4(d) and 4(e)).

### 3.5. MFXD Changed the Ratio of Th17 and Treg Cells in PBMCs

**Figure 5:**
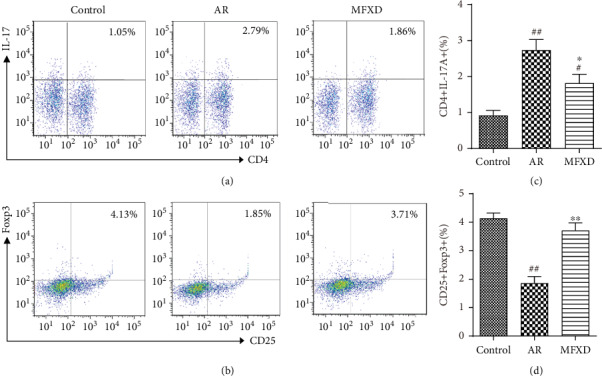
MFXD treatment maintained the percentages of Th17 and Treg cells in peripheral blood mononuclear cells (PBMCs) of AR rats. Representative flow cytometry dot plots for each group; the plots indicate the percentage of CD4+IL-17+ Th17 (a) and CD25+Foxp3+ Treg cells (b) among PBMCs. Percentages of CD4+IL-17+ Th17 cells (c) and CD25+Foxp3+ Treg cells (d) in each group. Data are presented as the means ± SD. ^#^*P* < 0.05 and ^##^*P* < 0.01 vs. the control group; ^∗^*P* < 0.05 and ^∗∗^*P* < 0.01 vs. the AR group.

As shown in Figure 5, the percentage of CD4+IL-17+ Th17 cells in the AR group was higher than that in the control group. By contrast, this percentage significantly decreased after MFXD treatment. The percentage of CD25+Foxp3+ Treg cells in the AR group was lower than that in the control group. However, the percentage of CD25+Foxp3+ Treg cells was elevated after administration with MFXD.

### 3.6. MFXD Regulated the Protein Expression of ROR*γ*t and Foxp3 in the Colon

**Figure 6:**
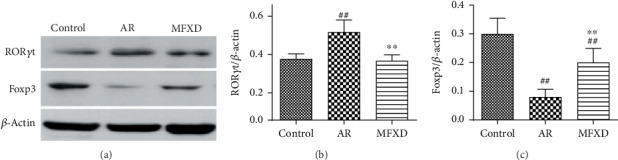
MFXD treatment regulated the protein expression of ROR*γ*t and Foxp3 in the colons of AR rats. (a) Western blotting results of ROR*γ*t, Foxp3, and *β*-actin in colonic homogenates of AR rats. The relative expression of ROR*γ*t (b) and Foxp3 (c) proteins. Data are presented as the means ± SD. ^#^*P* < 0.05 and ^##^*P* < 0.01 vs. the control group; ^∗^*P* < 0.05 and ^∗∗^*P* < 0.01 vs. the AR group.

Because the differentiation of CD4+ T cells to Th17 or Treg cells was mainly regulated by nuclear transcription factors, we further assessed changes in the expression of Foxp3 and ROR*γ*t by western blotting (Figure 6(a)). In agreement with the IL-17 and IL-10 data shown above, MFXD reduced the expression of ROR*γ*t (Figure 6(b), *P* < 0.01), but promoted the expression of Foxp3, compared with that in the AR group (Figure 6(c), *P* < 0.01).

## 4. Discussion

Although the therapeutic effect of MFXD on AR has been confirmed in clinical research, its mechanism of action remains elusive. In the present study, we showed that MFXD improved AR symptoms in rats and that this effect was associated with the regulation of gut microbiota structure and the balance of Th17/Treg cells. Herbal medicines can have a risk of adverse effects if not properly used [22]. The toxicity of herbal preparation may be attributed mainly to the inherent toxicity of plant and malpractice. Herbal medicines were processed into decocting pieces before being used. In the preparation of decoction, the toxicity of Fuzi and Xixin can be reduced by long time decocting. According to the records of *Shanghan Lun*, the experimental dose was determined by the conversion of body surface area. These measures ensured the safety of MFXD and avoided the occurrence of side effects.

AR is a common respiratory allergic disease, in which gut microbiota has been known as a key factor [23]. Chao and Shannon indexes were important *α*-diversity indicators of gut microbiota. The Chao index is used to estimate the species richness information of samples. The Shannon index reflects the diversity of species as a comprehensive reflection of species structure. The larger the value, the higher the diversity of gut microbiota. In present study, we noted that the Chao and Shannon indexes were significantly decreased in the AR group. This was consistent with epidemiological observations that low gut bacterial diversity during infancy augments the risk of allergic diseases [24]. However, MFXD treatment restored *α*-diversity, indicating that MFXD increased the richness and evenness of gut microbiota. Moreover, PCA and PCoA showed that the AR group was distinguished from the control group, suggesting that AR altered gut microbial composition. MFXD improved this pathological change due to AR to the normal condition. These results suggested that MFXD as a treatment of AR altered the structure of gut microbiota.

In the present study, MFXD increased the abundance of Firmicutes, Bacteroidetes, and Actinobacteria and decreased that of Proteobacteria and Cyanobacteria at the phylum levels, suggesting that MFXD regulated the composition of gut microbiota. Our results implied that MFXD modulates gut microbiota which may benefit in regulating Th17/Treg balance and improving the AR. Gut microbiota is necessary for the expansion and differentiation of intraintestinal and systemic immune cells, which regulates the host immune. For example, Th17 cells are produced by colonization of *Escherichia coli* and *Bacteroides fragilis.* Tregs can be induced by *Lactobacillus* and *Streptococcus strains* in the intestinal lamina propria [25]. A previous study found a decrease in the *Lachnospiraceae* and *Ruminococcaceae* and an increase in the *Enterobacteriaceae* which are correlated with the Treg/Th17 imbalance [26]. In our study, MFXD increased the contents of *Lactobacillus group* and *Ruminococcaceae* and decreased the levels of *Bacteroides fragilis* and Enterococcus spp., which indicated MFXD modulates the abundance of gut microbiota related to the differentiation of Th17 and Treg cells. Moreover, MFXD markedly increased the relative abundance of *Anaerotruncus*, *Butyricicoccus*, *Lachnospiraceae*, and *Ruminococcaceae*, which are bacteria capable of fermenting carbohydrates, degrading oligosaccharides, and producing SCFAs [27]. This is supported by the above finding that MFXD treatment increased the abundance of Firmicutes, which is a phylum composed of major SCFA-producing bacteria [28, 29]. These results implied that deficiency of SCFA-producing bacteria may be relative to AR development; however, MFXD restored the composition of gut microbiota and facilitated the abundance of SCFA-producing bacteria.

SCFAs mediate the communication between gut microbiota and the host immune system [30], regulate the balance and function of Th17/Treg, and maintain gut integrity and immune homeostasis, which affects the balance between pro- and anti-inflammatory cytokines in the body with AR [31, 32]. Thus, the levels of SCFAs in the feces and serum were detected. Our data found that MFXD treatment facilitated the production of SCFAs and increased their levels. Moreover, the number of Th17 and Treg cells in the blood is an important index of AR [33]. In this study, we found an increase in the proportion of Th17 cells and a decrease in the proportion of Treg cells in AR rats, which is consistent with the result of a previous study [34]. However, this change could be reversed by MFXD, and this reversion is mainly a reduction in the number of Th17 cells. AR is an allergic disease of the upper respiratory tract. The differentiation of Th17 may be related to the immune responses from the lung. Th17 are being generated from naive T cells by IL-1*β* and are expanded and stabilized further by IL-23 [35], which implied that the levels of these cytokines could reflect the differentiation of Th17. Results found that the increased levels of IL-1*β* and IL-23 in the lung could be decreased by the MFXD. Our results speculated that MFXD reversed the balance of Th17/Treg which may relate to the inhibition of Th17 differentiation.

ROR*γ*t and Foxp3 are transcription factors of Th17 and Treg, respectively, which impact their differentiation. Thus, we studied the expression of ROR*γ*t and Foxp3 in the colon. The data showed that the expression of ROR*γ*t and Foxp3 increased and decreased in AR rats, respectively, consistent with the result of a previous study [36], implying that Th17/Treg imbalance is linked to dysregulation of the transcription factors. Treatment with MFXD significantly improved these changes. Therefore, these results suggested that MFXD affected the differentiation, function, and stabilization of Th17 and Treg cells. Moreover, due to the importance of SCFAs on the AR, we further analyzed whether SCFAs alleviate the AR by distribution in the lung and impacting the Th17 differentiation. However, the concentrations of SCFAs in the lung tissue were very low, which cannot match the detection limit of GC-MS. These results may be explained as follows: (1) the serum levels of SCFAs were 1000 times lower than those in the fecal level (Figure 3). The low levels of SCFAs in the lung may be due to the metabolic degradation in the systemic circulation process; (2) MFXD regulated the differentiation of Th17 and Treg cells by SCFAs which may happen mainly in the intestine (Figure 6), rather than the SCFAs being absorbed into the blood and distributed in the lung. In this study, MFXD may inhibit the differentiation of Th17 by regulating the gut microbiota metabolite SCFAs in the intestine, which exerts its efficacy on the AR. However, how did the fecal SCFAs modulate the differentiation of Th17 during the treatment of MFXD, which is unclear and further study, warrants further exploration.

## 5. Conclusion

Our study showed that MFXD treatment improved the structure of gut microbiota, increased fecal SCFA content, and restored the balance of Th17/Treg, thereby protecting rats from AR. These findings have advanced our understanding of the mechanism underlying effect of MFXD on AR in terms of gut microbiota and immune regulation.

## Figures and Tables

**Table 1:** Relative abundance of bacteria at the genus level in each group (mean ± SD, *n* = 6).

Relative abundance (%)	Control	AR	MFXD
*Anaerotruncus*	1.17 ± 0.13	0.68 ± 0.05^##^	1.09 ± 0.14^∗∗^
*Bacteroides*	4.13 ± 0.49	6.65 ± 0.71^##^	4.36 ± 0.44^∗∗^
*Bifidobacterium*	0.03 ± 0.01	1.66 ± 0.56^##^	0.09 ± 0.06^∗∗^
*Butyricicoccus*	0.39 ± 0.10	0.11 ± 0.01^#^	0.45 ± 0.13^∗^
*Enterococcus*	0.07 ± 0.03	0.56 ± 0.16^##^	0.09 ± 0.00^∗∗^
*Erysipelotrichaceae*	0.03 ± 0.01	0.22 ± 0.08^#^	0.02 ± 0.01^∗^
*Lachnospiraceae*	23.64 ± 1.82	11.09 ± 0.76^##^	21.42 ± 0.19^∗∗^
*Ruminococcaceae*	5.27 ± 0.42	3.72 ± 0.27^##^	5.12 ± 0.57^∗∗^

^#^ *P* < 0.05 and ^##^*P* < 0.01 vs. the control group; ^∗^*P* < 0.05 and ^∗∗^*P* < 0.01 vs. the AR group.

**Table 2:** Bacterial groups quantified by qPCR (mean ± SD, *n* = 6).

Relative abundance (%)	Control	AR	MFXD
*Lactobacillus group*	8.2 ± 0.5	6.6 ± 0.7^##^	7.9 ± 0.5^∗∗^
*Bacteroides fragilis*	3.9 ± 0.5	4.9 ± 0.6^#^	4.0 ± 0.5^∗^
*Butyricicoccus pullicaecorum*	2.4 ± 0.3	1.8 ± 0.4^##^	2.1 ± 0.5^∗^
*Enterococcus spp.*	1.3 ± 0.3	2.4 ± 0.3^##^	1.5 ± 0.4^∗∗^

Note: bacterial copy number values were transformed into log10 bacteria per gram of stool. ^#^*P* < 0.05 and ^##^*P* < 0.01 vs. the control group; ^∗^*P* < 0.05 and ^∗∗^*P* < 0.01 vs. the AR group.

## Data Availability

The data used to support the findings of this study are available within the article and the supplementary materials, or from the authors upon reasonable request.

## Supplementary Materials

Supplementary Materials Table S1: symptom classification criteria of allergic rhinitis rats.
