# Supplementary material for: Mahuang Fuzi Xixin Decoction Ameliorates Allergic Rhinitis in Rats by Regulating the Gut Microbiota and Th17/Treg Balance
Source: J Immunol Res. 2020 May 25;2020:6841078. doi: 10.1155/2020/6841078 (PMC7267863; doi:10.1155/2020/6841078)
Supplement: Supplementary Materials — Table S1: symptom classification criteria of allergic rhinitis rats. [file 6841078.f1.docx]

Table S1 Symptom Classification Criteria of Allergic Rhinitis Rats

|  | Mild | Moderate | Severe |
| --- | --- | --- | --- |
| Scratching | mild | intermittent | persistent |
| Rhinorrhea | Small amount of nasal mucus | Columella nasi | Nostrils full of snot |
| Sneezing | ＜4 time/30 min | 4~10 time/30 min | ＞10 time/30 min |
| Score | 1 | 2 | 3 |
